# Transarterial chemoembolization for hepatocellular carcinoma with portal vein tumor thrombus: a meta-analysis

**DOI:** 10.1186/1471-230X-13-60

**Published:** 2013-04-08

**Authors:** Tong-Chun Xue, Xiao-Ying Xie, Lan Zhang, Xin Yin, Bo-Heng Zhang, Zheng-Gang Ren

**Affiliations:** 1 Liver Cancer Institute, Zhongshan Hospital, Fudan University, 180 Fenglin Road, Shanghai, 200032, P.R. China; 2 Key Laboratory of Carcinogenesis and Cancer Invasion (Fudan University), Ministry of Education, Shanghai, P.R. China; 3 Department of Medical Statistics, Zhongshan Hospital, Fudan University, Shanghai, P.R. China

**Keywords:** Chemoembolization, Hepatocellular carcinoma, Portal vein, Embolus, Meta-analysis

## Abstract

**Background:**

Although transarterial chemoembolization (TACE) has been used extensively for advanced hepatocellular carcinoma (HCC) with portal vein tumor thrombus (PVTT), no consensus has been reached and an evidence base for practice is lacking. This meta-analysis evaluated the efficacy and safety of TACE for treatment of HCC with PVTT.

**Methods:**

Ovid Medline, EMBASE, Web of Knowledge, and Cochrane library databases were searched up to August 2012 for controlled trials assessing TACE in patients with PVTT. Data concerning the study design, characteristics of trials, and outcomes were extracted. Hazard ratio (HR) and 95% confidence interval (CI) were calculated using random effects models.

**Results:**

Eight controlled trials involving 1601 HCC patients were included. TACE significantly improved the 6-month (HR, 0.41; 95% CI: 0.32–0.53; z, 6.28; p = 0.000) and 1-year (HR, 0.44; 95% CI: 0.34–0.57; z, 6.22; p = 0.000) overall survival of patients with PVTT compared with conservative treatment. Subgroup analyses showed that TACE was significantly effective in HCC patients whether with main portal vein (MPV) obstruction or with segmental PVTT. Fatal complications were rare, even in patients with MPV obstruction. Temporary liver decompensation and postembolization syndrome occurred frequently. However, they could be treated successfully with conservative treatment.

**Conclusions:**

TACE, as a safe treatment, has potential for incurring a survival benefit for advanced HCC with PVTT, even with MPV obstruction. Further large randomized controlled trials may be needed to confirm this result.

## Background

Hepatocellular carcinoma (HCC) has one of the highest mortality rates for malignancies worldwide, particularly in Asian countries [1]. Although preliminary screening and diagnosis have allowed HCC patients to benefit from radical resection, transplantation, or radiofrequency ablation, tumors in some patients still progress rapidly because of local spreading or metastases, particularly in those with background cirrhosis. Therefore, overall survival (OS) still can not be acquired the encouraged improvement in most patients.

Portal vein invasion is an important survival prognostic factor for HCC. To date, some treatments have been used for portal vein tumor thrombus (PVTT), such as transarterial chemoembolization (TACE), radiation, and systematic chemotherapy, none of which has strong evidence-based support. According to Barcelona Clinic Liver Cancer (BCLC) staging [2], HCC patients with PVTT, or BCLC stage C, can only receive sorafenib target therapy [3]. However, for patients with advanced HCC, including vascular invasion or extrahepatic metastases, the median survival time with sorafenib is short – only 6.5 months in Asia [4]. In developing countries, such as China, economic conditions restrict the application of sorafenib in some patients. Therefore, consecutive TACE is still used to treat selective patients with PVTT.

As the therapeutic approach of choice for unresectable HCC, effects of TACE have been confirmed by some randomized controlled trials (RCTs). A meta-analysis of prospective randomized trials has shown that survival is improved after TACE for unresectable HCC with good liver function preservation [5]. Nevertheless, it is generally accepted that TACE is not recommended in cases of macroscopic portal vein invasion because of the potentially increased risk of liver failure. Therefore, most designed and reported TACE-related clinical trials have excluded patients with PVTT, particularly with main portal vein (MPV) obstruction. Recently, however, some prospective controlled trials have shown the survival benefits of TACE treatment in advanced HCC with PVTT [6,7]. Therefore, the clear effects and safety of TACE in these patients remain controversial.

This meta-analysis of controlled trials was performed to evaluate the effects of TACE treatment in patients with HCC and PVTT, including MPV obstruction.

## Methods

### Search strategy and selection criteria

We performed a search of Ovid Medline (from 1945 to “in press”), EMBASE, Web of Knowledge including SCIE (Science Citation Index Expanded) and CPCI-S (Conference Proceedings Citation Index – Science) from 1997 to date, and the Cochrane library up to August 2012. At first, the terms “hepatocellular carcinoma,” “liver cancer,” “hepatoma,” “transarterial chemoembolization,” “portal vein,” “thrombus,” and “clinical trials” were used. Only a few trials were available; therefore, the scope of the search was expanded. The terms “chemoembolization” and “portal vein” were mainly used. The references in the articles retrieved were also searched for relevant titles.

Prospective controlled trials concerning HCC patients with PVTT, including MPV obstruction, who received TACE or conservative treatment, were the first choice for inclusion. Retrospective controlled trials with arms including TACE and conservative treatment were also included. Each included study was approved by an ethics committee or institutional review board. Exclusion criteria were: (1) no access to full text for quality assessment and data extraction; (2) lack of study controls; and (3) case reports.

### Data extraction and quality assessment

Two investigators independently reviewed all potentially eligible studies and collected data on patient and study characteristics. The Newcastle–Ottawa Scale (NOS) was used to assess the study quality. The NOS uses two different tools for case–control and cohort studies and consists of three parameters of quality: selection (0–4 points), comparability (0–2 points), and outcome assessment (0–3 points). The maximum possible score is 9 points, representing the highest methodological study.

### Data synthesis and analysis

The 6-month and 1-year OS were assessed as the primary measures of treatment effect, using hazard ratio (HR) with 95% confidence interval (CI). The methods for incorporating summary time-to-event data into the meta-analysis were performed as described previously [8]. In addition to the analysis of OS, we also assessed the tumor response to TACE. Complete response was defined as no evidence of neoplastic disease at computed tomography at the end of the treatment. Partial response was defined as a reduction in total tumor size by >50%.

Pooled analyses were calculated using random-effect models. Sensitivity analyses were performed to determine the stability of the overall treatment effects. We excluded each study at a time to ensure that no single study would be solely responsible for the significance of any result. Statistical heterogeneity was measured using *I*^2^ statistics. Subgroup analysis and meta-regression analyses were conducted to explore and explain diversity (heterogeneity) among the results of different studies. All p values were two-tailed, and statistical significance was set at 0.05. Statistical analyses were performed using STATA version 12.0 (StataCorp, College Station, TX, USA).

## Results

### Eligible studies

**Figure 1:**
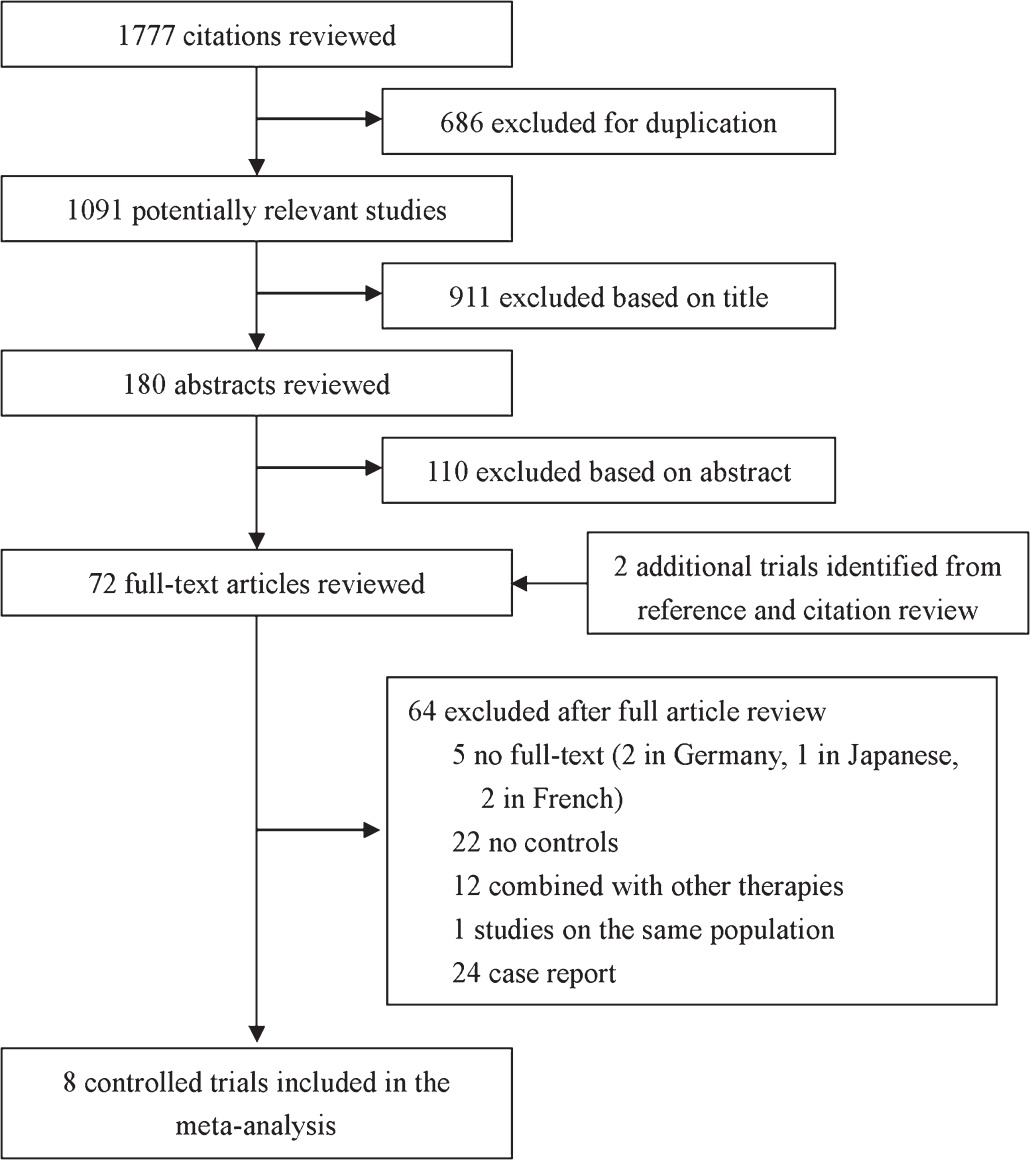
Search flow diagram for studies included in the meta-analysis.

The flow of selecting studies for the meta-analysis is shown in Figure 1. Among the initial 1777 hits, 72 articles were retrieved for detailed evaluation, and eight that satisfied the inclusion criteria were finally analyzed [6,7,9-14]. All eight trials were from Asia, including two from mainland China, one from Hong Kong, and five from South Korea. Four trials from Europe and another from Asia were not included because there was no full text. These excluded studies were reported before 1999. According to the available abstracts, it was difficult to recognize whether the specific designs comparing roles between TACE and conservative treatment for advanced HCC with PVTT were included. Quality assessment of the trials is shown in Additional file 1. Four studies were of high quality and another four had an NOS score of 6.

The main features of the eight studies included in the meta-analysis are shown in Table 1. Three trials were prospective [6,7,9], whereas the other five were retrospective. These trials included 1601 patients, 923 of whom received TACE. Two trials only included patients with MPV invasion [9,10]. In four trials, therapeutic regimens were compared with conservative treatment [6,7,9,10], and in the remaining four trials, treatment procedures were compared with each other. Two of the trials had more than three arms [11,12]. The percentage of male patients ranged from 84% to 100%, and the mean age from 45 to 55 years. Most patients had background hepatitis B virus (HBV). Patients who received TACE had higher Child–Pugh A/B ratio (1:1 to 4:1) than patients who received conservative treatment (1:2 to 1:1).

**Table 1:** Characteristics of clinical trials included in the meta-analysis

**Study and Treatment arm**	**Trial**	**Male (%)**	**Mean Age(y)**	**Etiology HBV/HCV**	**Cirrhosis**	**Child-Pugh A/B**	**Albumin (g/L)**
**Lee (Cancer 1997)**	P						
TACE(n=31)		84	52	29/2	NA	NA	37
Conservative(n=16)		81	51	13/2	NA	NA	36
**Luo (Ann Surg Oncol 2011)**	P						
TACE(n=84)		98	45	78/-	42	NA	41
Conservative(n=80)		91	47	70/-	35	NA	41
**Niu (Med Oncol 2011)**	P						
TACE(n=115)		93	46	106/-	NA	88/27	37
Conservative(n=35)		94	48	31/-	NA	21/14	36
**Chung (Radiology 2011)**	R						
TACE(n=83)		89	55	71/4	63	57/26	36
Conservative(n=42)		71	58	31/2	34	15/27	33
**KM Kim (JGH 2009)**	R						
TACE(n=149)		87	52	NA	NA	106/41	NA
TACI(n=53)		77	54	NA	NA	23/29	NA
Hepatic resection (n=19)		95	50	NA	NA	17/2	NA
Conservative(n=60)		90	53	NA	NA	18/33	NA
**Zhou (APJCP 2011)**	R						
TACE(n=10)		100	NA	12/-	10	5/5	NA
LT(n=12)		100	NA	10/-	9	4/6	NA
Hepatic resection (n=69)*		96	NA	66/-	63	55/14	NA
Conservative(n=30)		87	NA	26/-	23	9/11	NA
**JH Kim (APT 2009)**	R						
TACE(n=49)		90	54	37/2	36	30/17	NA
TACI(n=61)		87	54	56/1	45	22/32	NA
**Peng (Cancer 2012)**	R						
TACE(n=402)		93	55	356/7	363	389/13	34
Hepatic resection (n=201)		93	55	172/4	176	197/4	37

Note: *, Two subgroups of hepatic resection with other treatments were combined. *P*, perspective; *R*, retrospective; *LT*: liver transplantation; *NA*: not available.

The main variables in the TACE protocol were dose and type of chemotherapeutic agents. The mean number of TACE sessions was 1.5–3, and the highest was 14. TACE was repeated at fixed intervals of 1–3 months. The maximum amount of iodized oil was no more than 25 ml. The embolizing agent administered was gelatin sponge particles (Gelform) with or without mitomycin in all included trials. Nearly all TACE was performed in selective or super-selective style.

### OS

**Figure 2:**
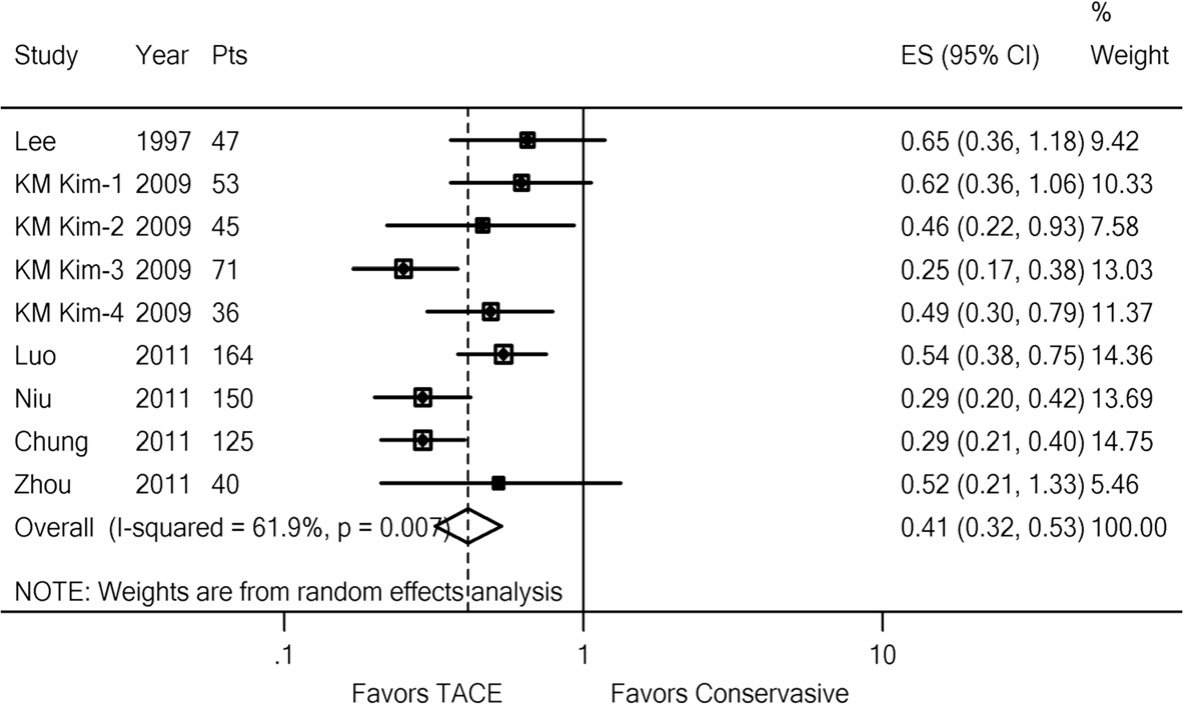
**Forest plot of the comparison between TACE and conservative treatment for 6-month OS.** A random effects model was used for HCC with PVTT. Each line represents an individual study result with the width of the horizontal line indicating 95% CI, the position of the box representing the point estimate, and the size of the box being proportional to the weight of the study. (KM Kim-1, 2: subgroup Child–Pugh A or Child–Pugh B in HCC with MPV invasion; KM Kim-3, 4: subgroup Child–Pugh A or Child–Pugh B in HCC with segmental PVTT).

We performed a meta-analysis of the six studies [6,7,9-12] in which chemoembolization was compared with conservative treatment for 6-month or 1-year OS. The effect of TACE on 6-month OS is shown in Figure 2 (six studies with nine comparisons: 731 patients). There was moderate statistical heterogeneity (*I*^2^ = 61.9%, p = 0.007). The 95% CI for the results of individual trials ranged widely. TACE had a favorable effect on survival in all six trials (nine comparisons). The pooled estimate of the TACE effect was significant (HR, 0.41; 95% CI: 0.32–0.53; z, 6.28; p = 0.000). Similarly, meta-analysis for 1-year OS confirmed the effect of TACE in patients with PVTT (Additional file 2). The pooled estimate was significant (HR, 0.44; 95% CI: 0.34–0.57; z, 6.22; p = 0.000).

Sensitivity analyses suggested that the favorable effect of TACE on overall 6-month survival was not affected following sequential exclusion of each study in turn (Additional file 3). Also, no trial affected the pooled effect of TACE on overall 1-year survival when it was omitted (Additional file 4).

**Figure 3:**
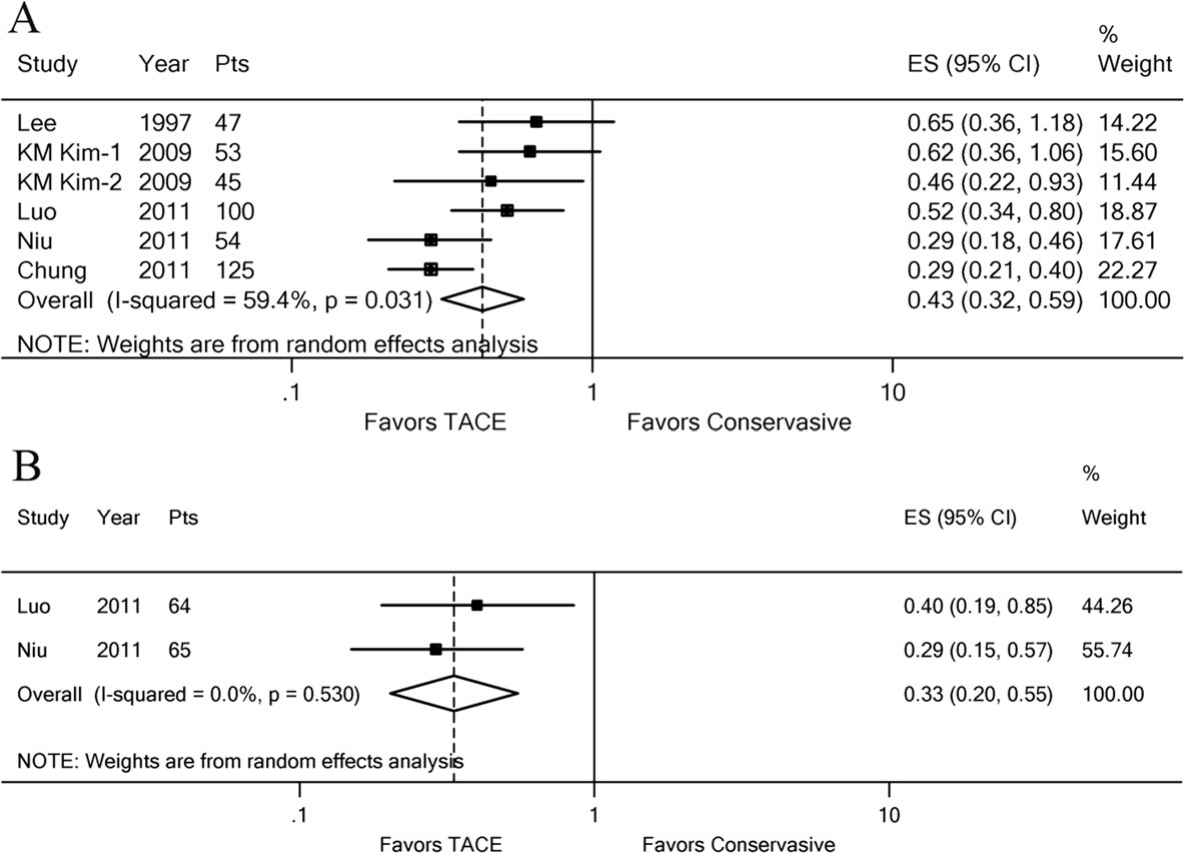
**Forest plots of the favored effect of TACE for 6-month OS.** The plots were based on the degree of portal vein invasion. (**A**) Subgroup analysis in HCC with MPV. (**B**) Subgroup analysis in HCC with segmental PVTT. Each line represents an individual study result with the width of the horizontal line indicating 95% CI, the position of the box representing the point estimate, and the size of the box being proportional to the weight of the study. (KM Kim-1, 2: subgroup Child–Pugh A or Child–Pugh B in HCC with MPV invasion).

We explored further the potential causes of the heterogeneity in the meta-analysis. First, we analyzed the effect of TACE on patients with MPV or segmental PVTT separately. Five trials (six comparisons) [6,7,9-11] included a comparison between TACE and conservative treatment. The effect of TACE on 6-month OS was favored in all five trials, and the pooled estimate of the TACE was significant (HR, 0.43; 95% CI: 0.32–0.59; z, 5.28; p = 0.000) (Figure 3). Statistical heterogeneity was seen (*I*^2^ = 59.4%, p = 0.031) in the subgroup with MPV obstruction (Figure 3A), but not in the subgroup with segmental PVTT (*I*^2^ = 0.0%, p = 0.530) (Figure 3B). The effect of TACE on 1-year OS showed similar results (Additional file 5). Therefore, the main cause of heterogeneity may lie in the subgroup with MPV obstruction.

**Figure 4:**
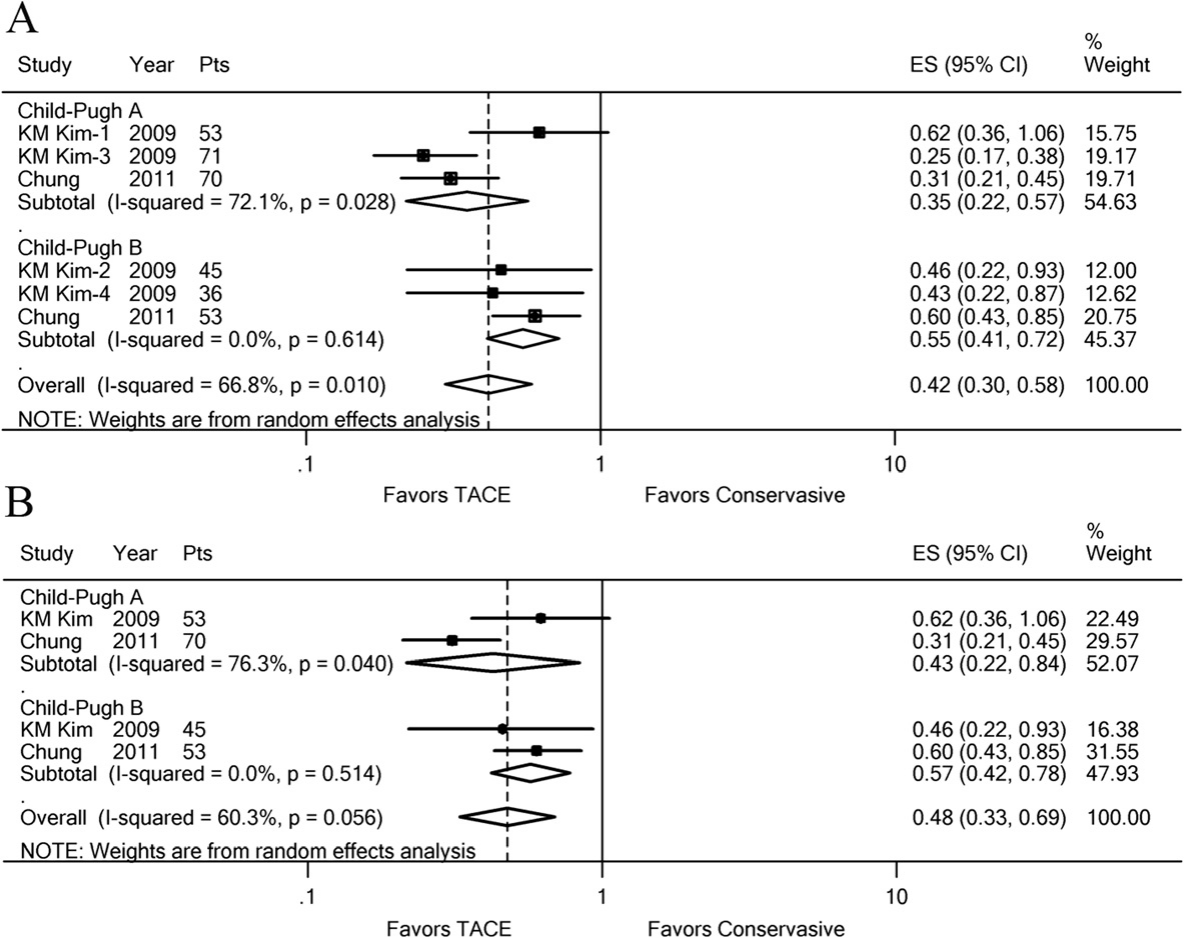
**Forest plots of the favored effect of TACE for 6-month OS.** The plot was based on the liver function, Child–Pugh A or B. (**A**) Subgroup analysis in HCC with PVTT. (**B**) Subgroup analysis in HCC with MPV invasion only. Each line represents an individual study result with the width of the horizontal line indicating 95% CI, the position of the box representing the point estimate, and the size of the box being proportional to the weight of the study. (KM Kim-1, 3: subgroup MPV invasion or segmental PVTT in HCC with Child–Pugh A; KM Kim-2, 4: subgroup MPV invasion or segmental PVTT in HCC with Child–Pugh B).

The trials suggested that liver function was a significant prognostic factor; therefore, we further analyzed the effect of TACE on patients with Child–Pugh A or B, separately. Only two trials [10,11] yielded related survival data. The pooled overall estimate favored TACE in patients with PVTT including MPV obstruction (HR, 0.42; 95% CI: 0.30–0.58; z, 4.94; p = 0.000), and the results suggested heterogeneity in the Child–Pugh A subgroup, but not in the Child–Pugh B group (Figure 4A). Analyzing the data from patients with MPV obstruction in two trials suggested a favorable effect of TACE in the Child–Pugh A subgroup (HR, 0.43; 95% CI: 0.22–0.84; z, 2.47; p = 0.014) and Child–Pugh B subgroup (HR, 0.57; 95% CI: 0.42–0.78; z, 3.56; p = 0.000) (Figure 4B). The *I*^2^ statistics in the Child–Pugh A subgroup increased to 76.3% (p = 0.040), which suggested that the main cause of heterogeneity was from patients with MPV obstruction and Child–Pugh class A. The effect of TACE on 1-year OS showed similar results (Additional file 6).

Meta-regression analysis was also used to explore the heterogeneity in trials for 6-month or 1-year OS. A total of seven trials (10 comparisons) were analyzed, which included one new trial that compared the effect of TACE and transarterial chemoinfusion (TACI). Year, trial type (perspective or retrospective), and country were the three features examined [6,7,9-13]. Mean age, proportion of male patients, percentage of HBV and MPV were also examined. Data about Child–Pugh class and diffuse tumor could not be analyzed because of incomplete data. Adjusted *R*^2^ values suggested that year and percentage of HBV partly explained the heterogeneity (13.2% and 3.3%, respectively).

### TACE and TACI or hepatic resection

**Figure 5:**
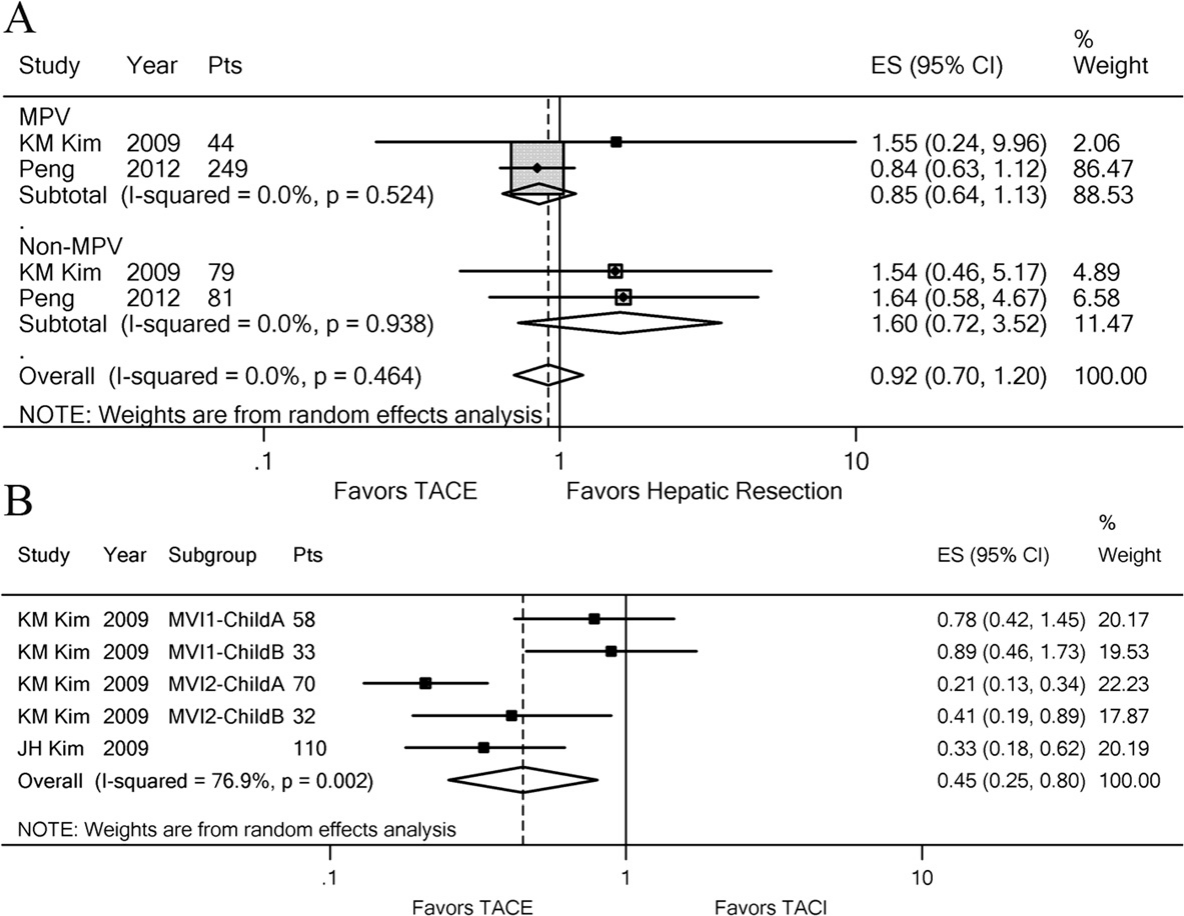
**Forest plots of the comparison between TACE and hepatic resection or TACI.** A random effects model was used for HCC with PVTT. (**A**) Subgroup analysis between TACE and hepatic resection for 1-year OS based on MPV invasion. (**B**) Comparison between TACE and TACI for 6-month OS. Each line represents an individual study result with the width of the horizontal line indicating 95% CI, the position of the box representing the point estimate, and the size of the box being proportional to the weight of the study.

We also evaluated whether there was evidence of different treatment effects based on comparisons between TACE and TACI or hepatic resection. Although the pooled estimate seemed to favor TACE, there was no significant treatment difference between TACE and hepatic resection [11,14] for 1-year OS (HR, 0.92; 95% CI: 0.70–1.20; z, 0.65; p = 0.519) (Figure 5A). Sensitivity analysis suggested that this beneficial effect was concealed when we omitted the MPV group in the study by Peng et al. [14] (p = 0.035). Subgroup analyses showed that TACE seemed to be more suitable for patients with MPV obstruction and hepatic resection for patients without MPV obstruction (Figure 5A). There was no evidence of statistical heterogeneity (*I*^2^ = 0.0%, p = 0.464). The difference between TACE and HR for 1-year OS was not significant (p = 0.519) (Additional file 7A). The pooled estimate of the TACE effect was significant when compared with TACI for 6-month OS (HR, 0.45; 95% CI: 0.25–0.80; z, 2.73; p = 0.006) (Figure 5B). Similarly, meta-analysis for 1-year OS also favored TACE in patients with PVTT (Additional file 7B).

### Tumor response

Most patients in the trials had diffuse HCC; therefore, the ability to measure changes in tumor size after treatment was limited. Data about tumor responses were reported in only two studies [11,14], which involved 489 patients. In the two trials, there were no complete responses. Between 17% [14] and 20% [11] of patients had a partial response. Stable disease was observed in 47% of patients in both trials. The rate of progressive disease ranged from 37% [11] to 38% [14].

Two trials [9,10] evaluated iodized oil (Lipidol) uptake by MPV. The presence of iodized oil uptake in the tumor thrombi was about 80% in nodular-type HCC and only 21% in diffuse-type [9]. Another study showed that the presence of iodized oil uptake in the MPV after TACE was a significant favorable prognostic factor [10].

### Treatment safety

The most frequent complication was postembolization syndrome, including fever, nausea, vomiting, or abdominal pain, with a rate ranging from 35% [13] to 94% [6]. One study [9] reported 65% fever and 65% abdominal pain, and another [14] reported 52% fever, 56% abdominal pain, and 49% vomiting. Temporary liver decompensation was observed in 26% [9] to 85% [6] of patients. However, <2% of patients had acute liver failure [9,10,14]. The rate of hyperbilirubinemia ranged from 2% [13] to 10% [9]. Inflammation was reported in three trials, including 2% with cholecystitis [6,14] and 0.2% [14] to 1% [10] with spontaneous bacterial peritonitis. Severe gastrointestinal bleeding was observed in 0–6% of patients [6,10,14], which was significantly less than the 17% [10] in patients with conservative treatment. Paralytic ileus occurred in 3% [9] to 5% [10] of patients. TACE-related deaths ranged from 0 [6] to 6% [13].

## Discussion

This meta-analysis showed that TACE was potentially suitable and safe for advanced HCC with PVTT, including patients with MPV obstruction. The beneficial effects were demonstrated by improved 6-month and 1-year OS in the TACE group, and by the improved tumor response when compared with conservative treatment. Moreover, the potential inhibition of tumor growth and spread by iodized oil uptake in the MPV after TACE may be pertinent to the OS benefits. The rate of fatal complications was low, even in patients with MPV obstruction. Although most patients had background cirrhosis, acute liver failure and gastrointestinal bleeding were rare. In contrast, patients who received conservative treatment were more prone to gastrointestinal bleeding, which may be related to rapid tumor progression. Temporary liver decompensation and postembolization syndrome occurred more frequently, which can be treated successfully with conservative treatment.

HCC is distributed unevenly worldwide, and morbidity and mortality are particularly high in Asia, including China, Japan, and Korea. According to the American Association for the Study of Liver Diseases (AASLD) guidelines for HCC treatment, advanced HCC with PVTT can only be treated with sorafenib-targeted therapy [3]. MPV obstruction is recognized as a complete contraindication. However, in most Asian countries, TACE is still used routinely for patients with PVTT. Consensus has been reached recently based on the guidelines from the main Asian countries with high HCC morbidity [15]. However, a clear evidence base for TACE in patients with PVTT is still lacking. The present meta-analysis indicated that TACE was a safe choice for advanced HCC with PVTT. Combination with improved super-selection techniques and modified methods such as DEB-TACE (doxorubicin-eluting bead transarterial chemoembolization) [16] may improve the effects and safety of TACE. For selected patients with MPV obstruction, especially those with established collateral circulation and good liver function preservation, TACE treatment may prolong survival.

Subgroup estimation indicated that TACE was effective for improving survival in patients with MPV invasion or segmental PVTT. The main cause of heterogeneity may lie in the MPV subgroup, particularly in those with Child–Pugh class A. This meta-regression analysis could only find a minor cause of heterogeneity. We think that part of the reason is the limited data, such as liver function, and the number of available trials. The variables in the TACE protocol such as dose and type of chemotherapeutic agents may have caused heterogeneity. Also, type of tumor (diffuse or nodular) may be another important reason, because diffuse-type HCC was more prone to develop MPV obstruction than nodular-type.

Sorafenib is recognized as the standard therapy for advanced HCC with PVTT or metastasis. In Asia, the median survival of patients receiving sorafenib is 6.5 months. However, trials comparing TACE and sorafenib for patients with PVTT have been rare. The latest study to compare the efficacy of TACE and sorafenib in patients with advanced-stage (BCLC stage C) HCC suggests a promising outcome with TACE [17]. Combined treatment with TACE and sorafenib also indicated the survival benefits for selective patients [18,19]. In this meta-analysis, our exploratory analyses indicated that TACE was better than TACI for patients with PVTT. When compared with hepatic resection, there was no significant difference for improving survival. It seems that TACE was more suitable for patients with MPV invasion, whereas hepatic resection was more suitable for the PVTT group.

### Study limitations

Drawbacks pertinent to this meta-analysis were mainly the differences in study characteristics among the included studies. The included trials were nonrandomized trials, partly because of great concern about the potential risk of hepatic failure after TACE. Also, the patient selection for different treatments was biased. Patients with better liver function tended to be selected into the TACE group, whereas those with poorer liver function may have been willing to receive conservative treatment. Therefore, truly randomized trials are difficult to design and perform. As a result of limited eligible trials, five retrospective trials were also included in this meta-analysis. All the trials included in this meta-analysis were from Asia, which is the highest-risk area for HCC. Four studies from Europe were excluded because of the unavailability of full text. Therefore, it is worth performing more large RCTs, even multicenter studies, to confirm the effect of TACE on HCC with PVTT.

## Conclusions

In spite of differences in study design and population characteristics, the meta-analysis demonstrated that TACE has potential to improve survival and is safe for advanced HCC with PVTT, even with MPV obstruction. Further well-designed controlled trials may be needed to confirm this effect.

## Abbreviations

HCC: Hepatocellular carcinoma; OS: Overall survival; PVTT: Portal vein tumor thrombus; TACE: Transarterial chemoembolization; RCT: Randomized controlled trial; MPV: Main portal vein; NOS: Newcastle-Ottawa Scale; HR: Hazard ratios; CI: Confidence interval; HBV: Hepatitis B virus; TACI: Transarterial chemoinfusion

## Competing interests

The authors declare that they have no competing interest.

## Authors’ contributions

Dr. TC Xue participated in study conception and design, data acquisition, data analysis and interpretation, statistical analysis, and drafting the manuscript. Dr. XY Xie, Dr. L Zhang, and Dr X Yin participated in the statistical analysis and helped to draft the manuscript. Dr. BH Zhang carried out the statistical analysis. Prof. ZG Ren participated in study design. All authors read and approved the final manuscript.

## Pre-publication history

The pre-publication history for this paper can be accessed here:

http://www.biomedcentral.com/1471-230X/13/60/prepub

## Supplementary Material

Additional file 1: Table S1 Quality assessment of included eight trials according to the Newcastle-Ottawa scale. Click here for file

Additional file 2 **Forest plot of the comparison between TACE and conservative treatment for 1-year OS.** A random effects model was used for HCC with PVTT. Each line represents an individual study result with the width of the horizontal line indicating 95% CI, the position of the box representing the point estimate, and the size of the box being proportional to the weight of the study. (KM Kim-1, 2: subgroup Child–Pugh A or Child–Pugh B in HCC with MPV invasion; KM Kim-3, 4: subgroup Child–Pugh A or Child–Pugh B in HCC with segmental PVTT). Click here for file

Additional file 3 **Sensitivity analyses of the favored effect of TACE for 6-month OS.** The analyses were carried out by a sequential exclusion of each study in turn. (KM Kim-1, 2: subgroup Child–Pugh A or Child–Pugh B in HCC with MPV invasion; KM Kim-3, 4: subgroup Child–Pugh A or Child–Pugh B in HCC with segmental PVTT). Click here for file

Additional file 4 **Sensitivity analyses of the favored effect of TACE for 1-year OS.** The analyses were carried out by a sequential exclusion of each study in turn. (KM Kim-1, 2: subgroup Child–Pugh A or Child–Pugh B in HCC with MPV invasion; KM Kim-3, 4: subgroup Child–Pugh A or Child–Pugh B in HCC with segmental PVTT). (TIFF 75 kb) Click here for file

Additional file 5 **Forest plots of the favored effect of TACE for 1-year OS.** The plots were based on the degree of portal vein invasion. (**A**) Subgroup analysis in HCC with MPV. (**B**) Subgroup analysis in HCC with segmental PVTT. Each line represents an individual study result with the width of the horizontal line indicating 95% CI, the position of the box representing the point estimate, and the size of the box being proportional to the weight of the study. (KM Kim-1, 2: subgroup Child–Pugh A or Child–Pugh B in HCC with MPV invasion). Click here for file

Additional file 6: **Forest plots of the favored effect of TACE for 1-year OS.** The plots were based on the liver function, Child–Pugh A or B. (**A**) Subgroup analysis in HCC with PVTT. (**B**) Subgroup analysis in HCC with MPV invasion only. Each line represents an individual study result with the width of the horizontal line indicating 95% CI, the position of the box representing the point estimate, and the size of the box being proportional to the weight of the study. (KM Kim-1, 3: subgroup MPV invasion or segmental PVTT in HCC with Child–Pugh A; KM Kim-2, 4: subgroup MPV invasion or segmental PVTT in HCC with Child–Pugh B). Click here for file

Additional file 7 **Forest plots of the comparison between TACE and other treatments for 1-year OS.** A random effects model was used for HCC with PVTT. (**A**) Comparison between TACE and hepatic resection. (**B**) Comparison between TACE and TACI. Each line represents an individual study result with the width of the horizontal line indicating 95% CI, the position of the box representing the point estimate, and the size of the box being proportional to the weight of the study. Click here for file
