# Supplementary material for: Transarterial chemoembolization for hepatocellular carcinoma with portal vein tumor thrombus: a meta-analysis
Source: BMC Gastroenterol. 2013 Apr 8;13:60. doi: 10.1186/1471-230X-13-60 (PMC3626696; doi:10.1186/1471-230X-13-60)
Supplement: Additional file 1: Table S1 — Quality assessment of included eight trials according to the Newcastle-Ottawa scale. [file 1471-230X-13-60-S1.doc]

Supplemental Table 1 Quality assessment according to the Newcastle-Ottawa scale

| Study | Trial | Selection | Comparability | Outcome | Total |
| --- | --- | --- | --- | --- | --- |
| Lee (Cancer 1997) | Prospective | 4 | 1 | 2 | 7 |
| Luo (Ann Surg Oncol 2011) | Prospective | 4 | 1 | 2 | 7 |
| Niu (Med Oncol 2011) | Prospective | 4 | 1 | 2 | 7 |
| Chung (Radiology 2011) | Retrospective | 3 | 1 | 2 | 6 |
| KM Kim (JGH 2009) | Retrospective | 3 | 2 | 2 | 7 |
| Zhou (APJCP 2011) | Retrospective | 3 | 1 | 2 | 6 |
| JH Kim (APT 2009) | Retrospective | 3 | 1 | 2 | 6 |
| Peng (Cancer 2012) | Retrospective | 3 | 1 | 2 | 6 |
| Average |  | 3.4 | 1.1 | 2 | 6.5 |
