# Supplementary figures and images for: Transarterial chemoembolization for hepatocellular carcinoma with portal vein tumor thrombus: a meta-analysis
Source: BMC Gastroenterol. 2013 Apr 8;13:60. doi: 10.1186/1471-230X-13-60 (PMC3626696; doi:10.1186/1471-230X-13-60)

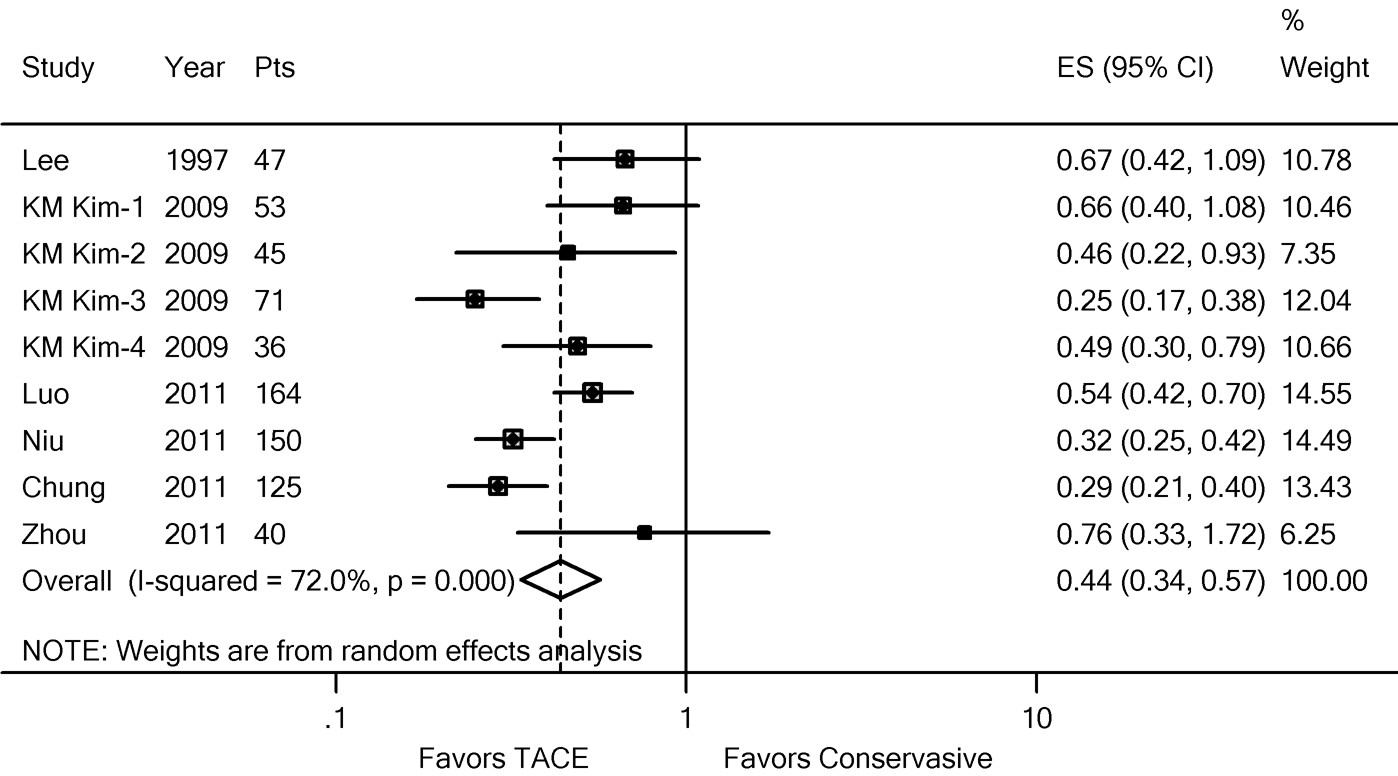

Supplement: Additional file 2 — Forest plot of the comparison between TACE and conservative treatment for 1-year OS. A random effects model was used for HCC with PVTT. Each line represents an individual study result with the width of the horizontal line indicating 95% CI, the position of the box representing the point estimate, and the size of the box being proportional to the weight of the study. (KM Kim-1, 2: subgroup Child–Pugh A or Child–Pugh B in HCC with MPV invasion; KM Kim-3, 4: subgroup Child–Pugh A or Child–Pugh B in HCC with segmental PVTT). [file 1471-230X-13-60-S2.tiff]

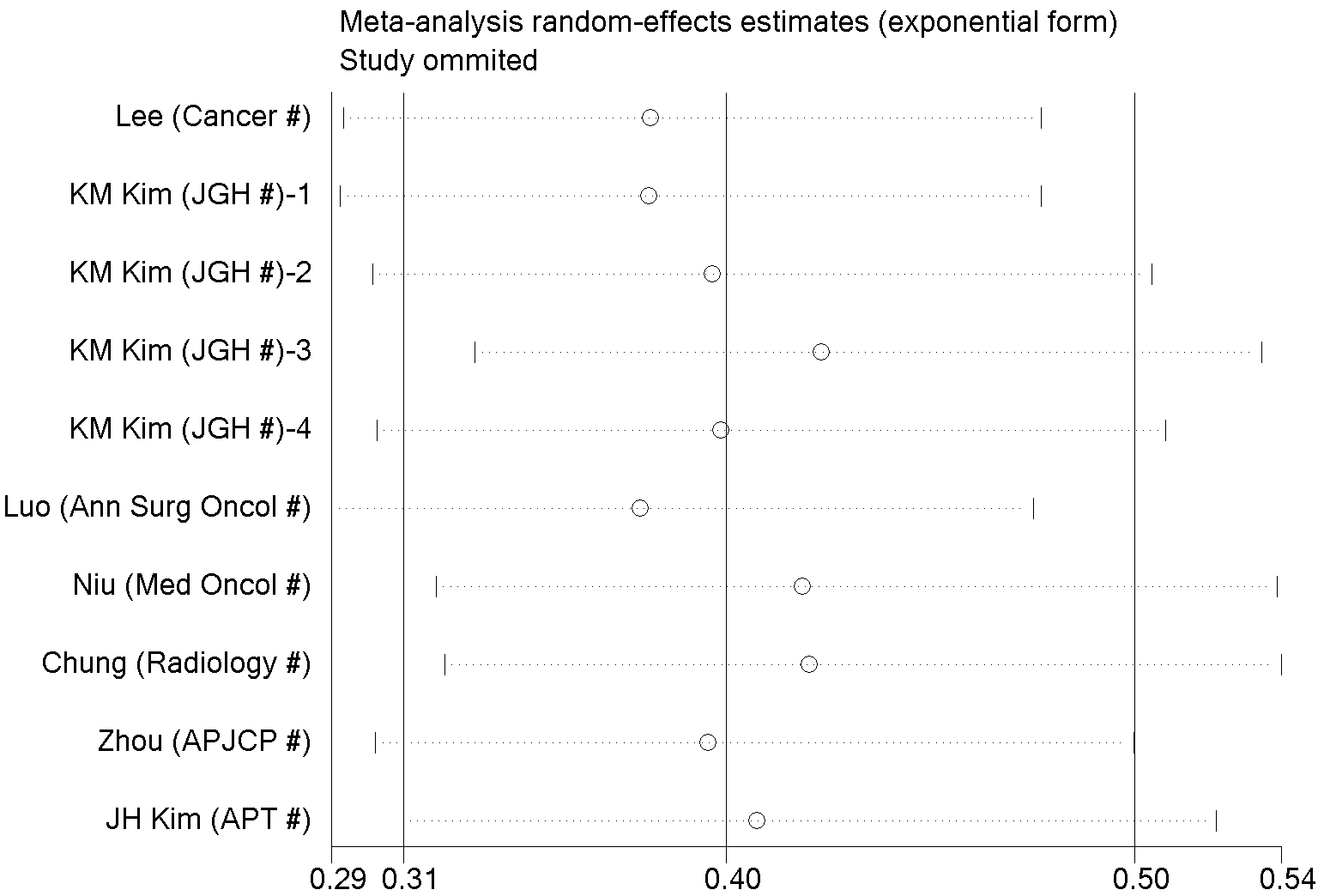

Supplement: Additional file 3 — Sensitivity analyses of the favored effect of TACE for 6-month OS. The analyses were carried out by a sequential exclusion of each study in turn. (KM Kim-1, 2: subgroup Child–Pugh A or Child–Pugh B in HCC with MPV invasion; KM Kim-3, 4: subgroup Child–Pugh A or Child–Pugh B in HCC with segmental PVTT). [file 1471-230X-13-60-S3.tiff]

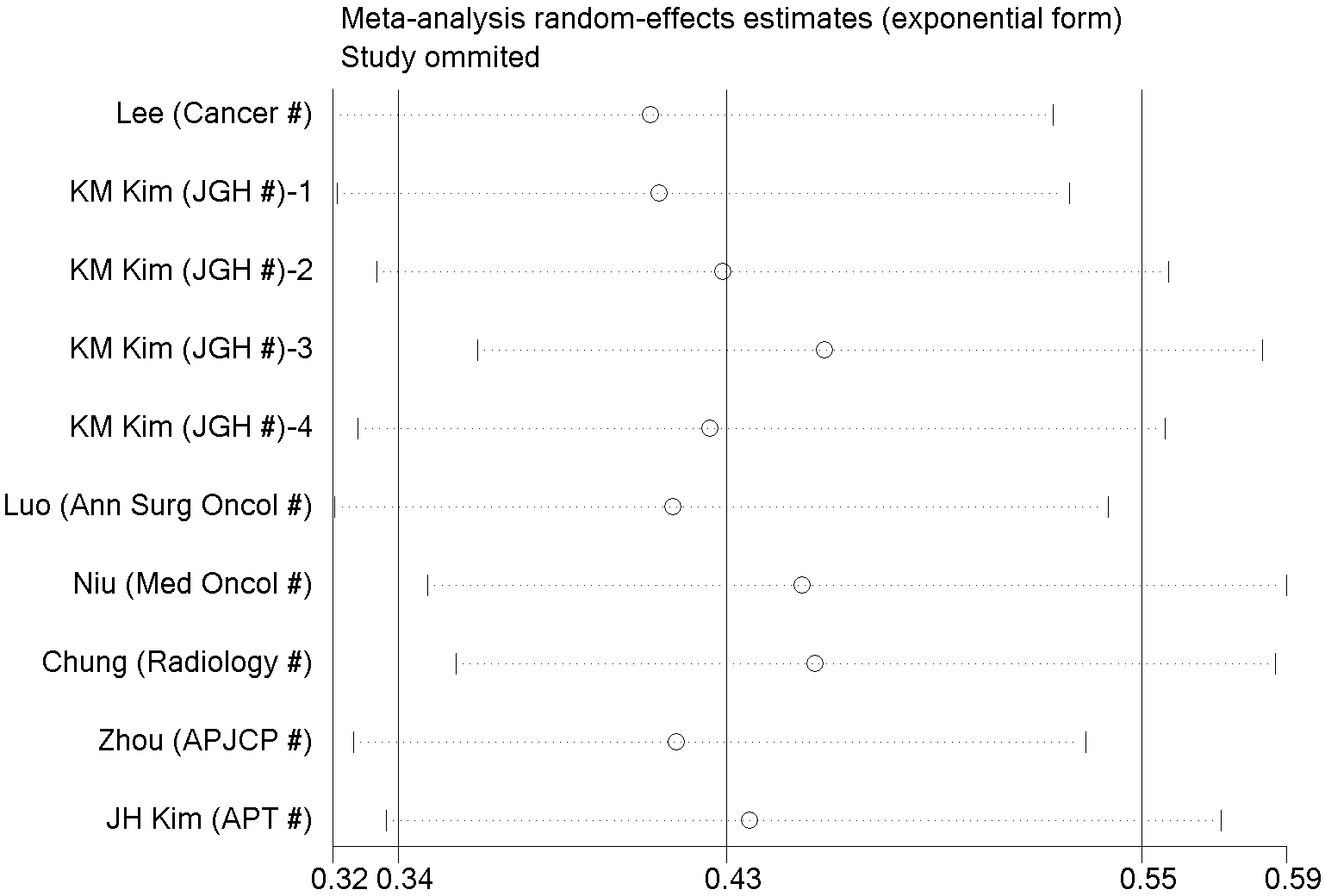

Supplement: Additional file 4 — Sensitivity analyses of the favored effect of TACE for 1-year OS. The analyses were carried out by a sequential exclusion of each study in turn. (KM Kim-1, 2: subgroup Child–Pugh A or Child–Pugh B in HCC with MPV invasion; KM Kim-3, 4: subgroup Child–Pugh A or Child–Pugh B in HCC with segmental PVTT). (TIFF 75 kb) [file 1471-230X-13-60-S4.tiff]

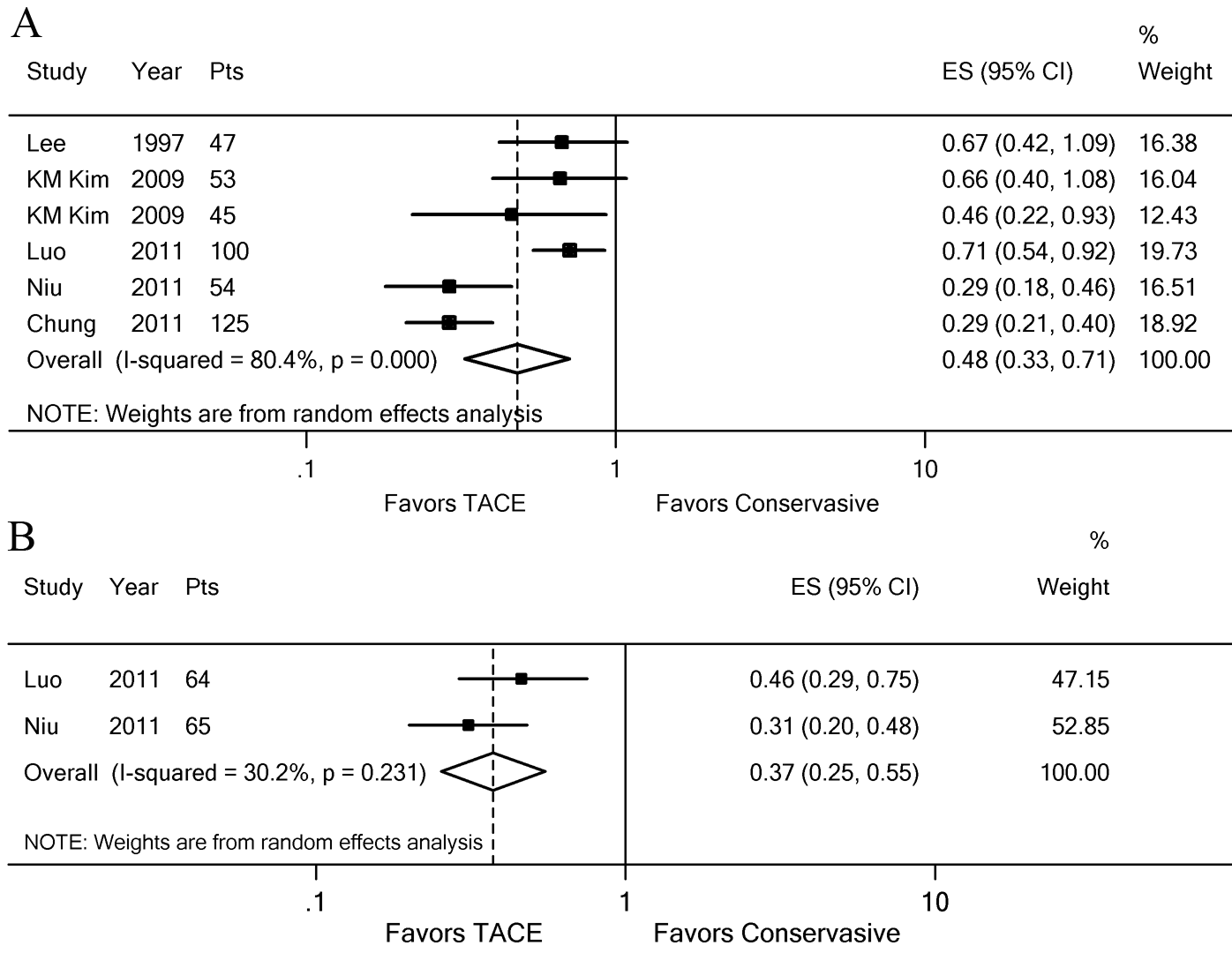

Supplement: Additional file 5 — Forest plots of the favored effect of TACE for 1-year OS. The plots were based on the degree of portal vein invasion. (A) Subgroup analysis in HCC with MPV. (B) Subgroup analysis in HCC with segmental PVTT. Each line represents an individual study result with the width of the horizontal line indicating 95% CI, the position of the box representing the point estimate, and the size of the box being proportional to the weight of the study. (KM Kim-1, 2: subgroup Child–Pugh A or Child–Pugh B in HCC with MPV invasion). [file 1471-230X-13-60-S5.tiff]

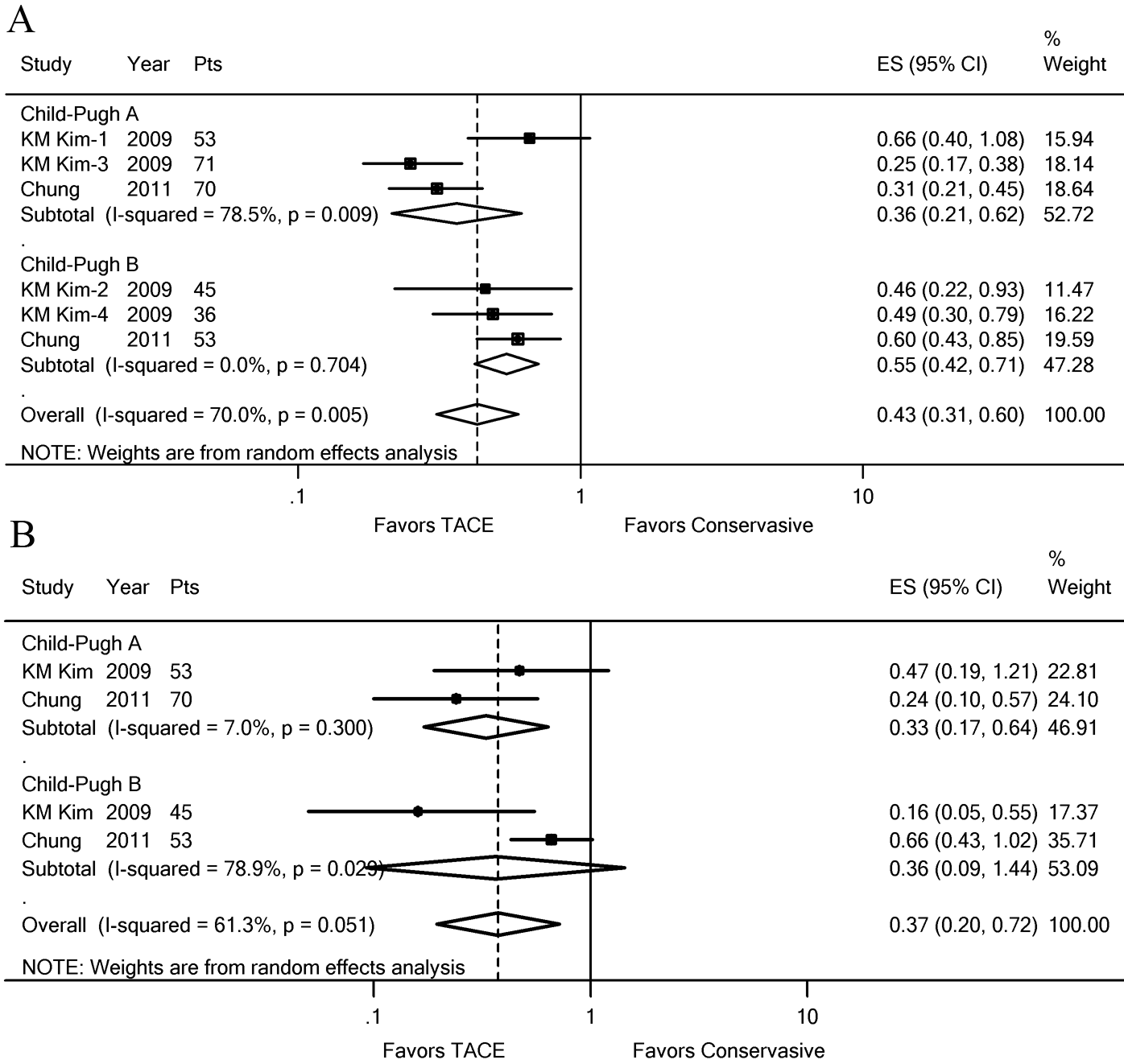

Supplement: Additional file 6: — Forest plots of the favored effect of TACE for 1-year OS. The plots were based on the liver function, Child–Pugh A or B. (A) Subgroup analysis in HCC with PVTT. (B) Subgroup analysis in HCC with MPV invasion only. Each line represents an individual study result with the width of the horizontal line indicating 95% CI, the position of the box representing the point estimate, and the size of the box being proportional to the weight of the study. (KM Kim-1, 3: subgroup MPV invasion or segmental PVTT in HCC with Child–Pugh A; KM Kim-2, 4: subgroup MPV invasion or segmental PVTT in HCC with Child–Pugh B). [file 1471-230X-13-60-S6.tiff]

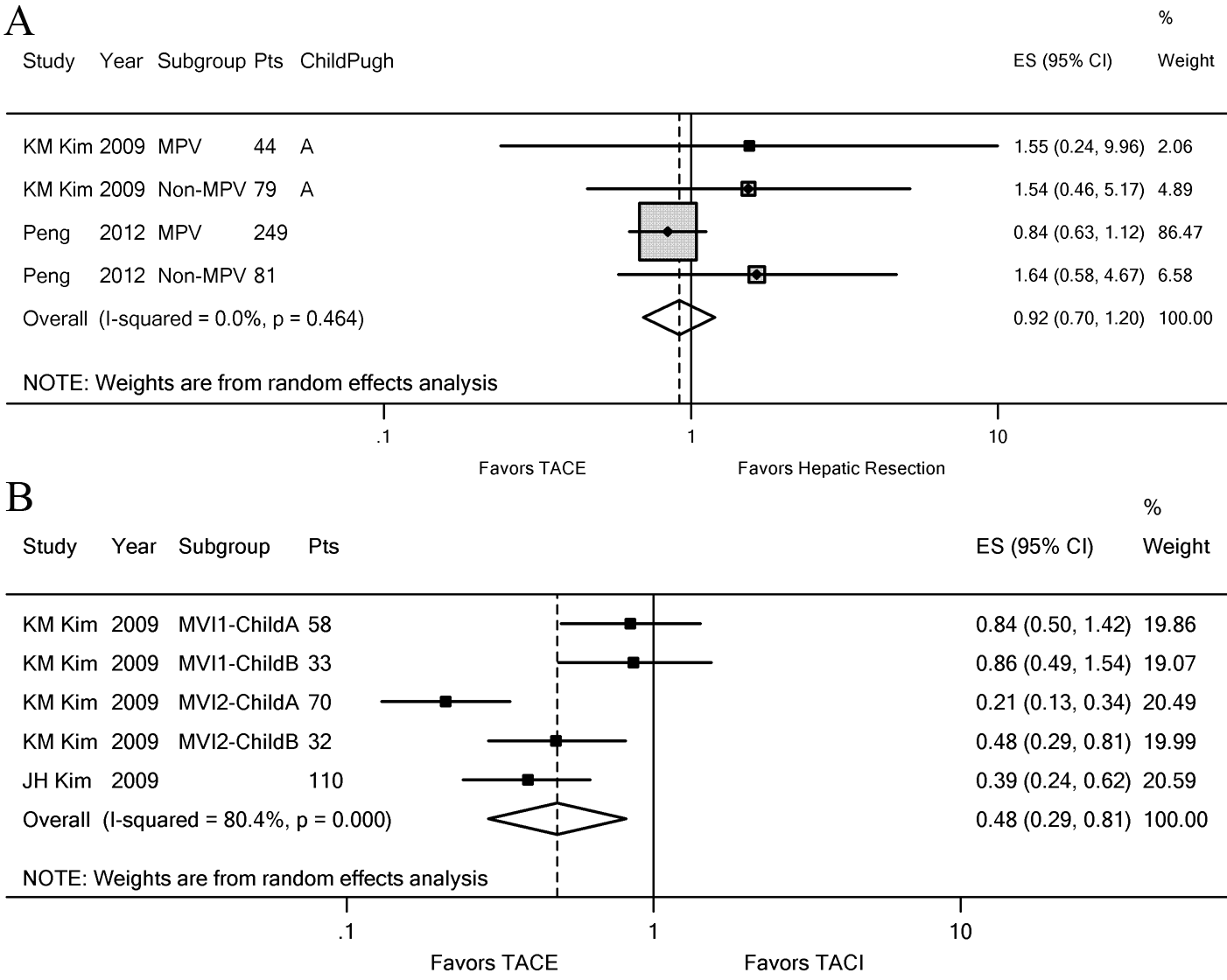

Supplement: Additional file 7 — Forest plots of the comparison between TACE and other treatments for 1-year OS. A random effects model was used for HCC with PVTT. (A) Comparison between TACE and hepatic resection. (B) Comparison between TACE and TACI. Each line represents an individual study result with the width of the horizontal line indicating 95% CI, the position of the box representing the point estimate, and the size of the box being proportional to the weight of the study. [file 1471-230X-13-60-S7.tiff]
